# Comparative analysis of response to treatments and molecular features of tumor-derived organoids *versus* cell lines and PDX derived from the same ovarian clear cell carcinoma

**DOI:** 10.1186/s13046-023-02809-8

**Published:** 2023-10-07

**Authors:** Lucie Thorel, Pierre-Marie Morice, Hippolyte Paysant, Romane Florent, Guillaume Babin, Cécilia Thomine, Marion Perréard, Edwige Abeilard, Florence Giffard, Emilie Brotin, Christophe Denoyelle, Céline Villenet, Shéhérazade Sebda, Mélanie Briand, Florence Joly, Enora Dolivet, Didier Goux, Cécile Blanc-Fournier, Corinne Jeanne, Marie Villedieu, Matthieu Meryet-Figuiere, Martin Figeac, Laurent Poulain, Louis-Bastien Weiswald

**Affiliations:** 1 Université de Caen Normandie, INSERM U1086 ANTICIPE (Interdisciplinary Research Unit for Cancers Prevention and Treatment), BioTICLA Laboratory (Precision Medicine for Ovarian Cancers), 3 Avenue du Général Harris, BP 45026, 14 076 Caen, Cedex 05 France; 2 Université de Caen Normandie, Services Unit PLATON, ORGAPRED Core Facility, Caen, France; 3 UNICANCER, Comprehensive Cancer Center François Baclesse, Caen, France; 4 UNICANCER, Comprehensive Cancer Center Francois Baclesse, Department of Surgery, Caen, France; 5 Université de Caen Normandie, Services Unit PLATON, ImpedanCell Core Facility, Caen, France; 6 University of Lille, CNRS, Inserm, CHU Lille, Institut Pasteur de Lille, US 41 - UAR 2014 - PLBS, Lille, France; 7 UNICANCER, Comprehensive Cancer Center Francois Baclesse, Biological Resources Center ‘OvaRessources’, Caen, France; 8 UNICANCER, Comprehensive Cancer Center Francois Baclesse, Clinical Research Department, Caen, France; 9 Université de Caen Normandie, Services Unit EMERODE, « Centre de Microscopie Appliquée À La Biologie » CMAbio3, Caen, France; 10 UNICANCER, Comprehensive Cancer Center Francois Baclesse, Department of Biopathology, Caen, France

**Keywords:** Ovarian clear cell carcinoma, Patient-derived tumor organoids, Patient-derived xenograft, Patient-derived cell lines, Precision medicine, Predictive functional assays

## Abstract

**Background:**

In the era of personalized medicine, the establishment of preclinical models of cancer that faithfully recapitulate original tumors is essential to potentially guide clinical decisions.

**Methods:**

We established 7 models [4 cell lines, 2 Patient-Derived Tumor Organoids (PDTO) and 1 Patient-Derived Xenograft (PDX)], all derived from the same Ovarian Clear Cell Carcinoma (OCCC). To determine the relevance of each of these models, comprehensive characterization was performed based on morphological, histological, and transcriptomic analyses as well as on the evaluation of their response to the treatments received by the patient. These results were compared to the clinical data.

**Results:**

Only the PDX and PDTO models derived from the patient tumor were able to recapitulate the patient tumor heterogeneity. The patient was refractory to carboplatin, doxorubicin and gemcitabine, while tumor cell lines were sensitive to these treatments. In contrast, PDX and PDTO models displayed resistance to the 3 drugs. The transcriptomic analysis was consistent with these results since the models recapitulating faithfully the clinical response grouped together away from the other classical 2D cell culture models. We next investigated the potential of drugs that have not been used in the patient clinical management and we identified the HDAC inhibitor belinostat as a potential effective treatment based on PDTO response.

**Conclusions:**

PDX and PDTO appear to be the most relevant models, but only PDTO seem to present all the necessary prerequisites for predictive purposes and could constitute relevant tools for therapeutic decision support in the context of these particularly aggressive cancers refractory to conventional treatments.

**Supplementary Information:**

The online version contains supplementary material available at 10.1186/s13046-023-02809-8.

## Background

Preclinical models of cancer are essential for understanding cancer biology and developing effective cancer therapeutics, and potentially to guide clinical decision-making in the era of personalized medicine. Therefore, it is required that these preclinical cancer models closely recapitulate original tumors, including histo- and molecular pathology, and match patient’s drug response.

Tumor cell lines grown as monolayer and mouse xenografts derived from those cells have been used for decades. The easy use, cost-effectiveness and ability to grow cells from many origins, made 2D culture as one of the most employed preclinical models. However, despite their invaluable contribution to cancer research and drug development, evidence indicate that in vitro cell lines diverge from the tumors from which they were derived, and consequently have a poor clinical predictive power [1]. Moreover, 2D-based monolayer cell culture systems are limited due to a lack of structural architecture. It is now well admitted that 3D cell–cell interactions and 3D chemical gradients (nutrients and oxygen) have a strong impact on many cellular processes including cell heterogeneity, proliferation, migration, invasion, cell structure, adhesion, mechanotransduction, and response to treatments [2]. In contrast, Patient-Derived tumor Xenografts (PDX) established from patient tumor fragments directly transplanted into immunodeficient mice recapitulate key patient tumor characteristics and demonstrate high concordance with clinical outcomes [3]. However, they are limited by the low success rate of establishment for some tumor types, the long time required for the establishment, the time-consuming and costly process of their use, as well as by their ethical issues [4].

As an intermediate model between in vitro cancer cell lines and in vivo tumors, the recent emergence of Patient-Derived Tumor Organoids (PDTO) models expands the repertoire of relevant 3D models. They are obtained from patient tumor cells embedded in basement membrane matrix and cultured in a medium supplemented with a cocktail of cell signaling pathways activators (growth factors) and inhibitors (pharmacological molecules) to mimic in vivo niche conditions and allow long term growth. PDTO faithfully reproduce the histological and molecular characteristics of the tumor from which they are derived. They can be rapidly expanded after tumor resection and can be established from a small sample size such as needle biopsy with a high success rate compared to other models [5]. More importantly, despite the lack of stromal cells, there are more and more evidence that the treatment response of PDTO is correlated to the clinical response [6, 7].

Functional precision medicine in oncology is an approach based on direct exposure of patient-derived tumor model to drugs to predict clinical response [8]. Improved feasibility of generating preclinical models representing the patient tumor has made these models accessible for personalized therapy. Functional predictive assays based on these models could predict patient response to conventional chemotherapy and/or select the most effective treatment among the approved therapies when several options are available. Alternatively, functional assays could help in the choice of addressing patients to clinical trials [9]. Nevertheless, it still remains to be determined whether functional assays can improve outcomes and eventually become standard tools in clinical oncology. In this context, it is essential to determine the choice of the most appropriate personalized tumor model. In order to make the best choice, the success rate of establishment, the delay between the surgery and the results of the functional assays, as well as the reliability of the results will have to be considered. Clinical trials are currently enrolling in order to evaluate the interest of functional predictive assays based on personalized tumor models (PDX, PDTO or ex vivo tumor cells) in oncology practice [8].

Ovarian cancer is the second cause of death among women with gynecological cancers around the world [10]. Epithelial ovarian cancers (EOC) represent 95% of ovarian cancers and are associated with a poor clinical outcome due to a late diagnosis (dissemination stage III and IV) and to their heterogenous nature. Among them, five major subtypes with distinct biological and molecular properties have been categorized by histology: high-grade serous, low-grade serous, clear cell, endometrioid, and mucinous ovarian carcinomas. Despite an initial good response of ovarian cancer patients to first-line treatment (usually consisting of surgical cytoreduction and platinum-taxane combination therapy), the majority of women with advanced-stage ovarian cancer will relapse. Ovarian clear cell carcinoma (OCCC) is characterized by the clear cytoplasmic appearance of tumor cells, due to an accumulation of glycogen. It is considered as a rare tumor type as defined by the Gynecologic Cancer Inter Group (GCIG) and represents approximately 5% of ovarian cancers in North America and Europe, but almost 25% of all patients with EOC in Japan [10]. OCCC has the worst prognosis amongst advanced EOC most likely due to poor response to platinum-based chemotherapy [11]. It is therefore crucial to develop relevant preclinical models of OCCC to identify new therapeutic strategies. Eventually, functional assays performed on such models could allow to predict clinical response to treatments and to guide therapeutic decision-making.

According to the comprehensive list of OCCC cell lines provided by Franklin et al. [12] and to the establishment of the 105C cell line [13], 29 OCCC cell lines have been established so far and some of them are able to generate tight spheroids when cultured in non-adherent conditions [14]. Nevertheless, most of them have not been extensively characterized and less than half of them have been deposited in cell banks. Regarding PDX and PDTO models, availability of these models is limited: only 2 PDTO models [15, 16] were established so far and Cybula et al. reported the establishment of 15 PDX of OCCC from 4 research groups [17]. Beyond these OCCC PDX models, a PDX from metastatic clear cell adenocarcinoma of Mullerian origin (cervix, endometrium, and fallopian tubes) was used to accurately predict resistance of the patient to first- and second-line therapies in a clinically workable timeframe as proof of concept of PDX-based functional predictive assay [18].

In this study, we described the first comprehensive comparative phenotypic and molecular analysis of PDX, PDTO and cell line models derived from the same OCCC patient tumor. We also investigated the correlation between the response of these tumor models and the clinical response to determine their interest for a potential use in oncology practice. Our study strongly suggests that PDTO are faithful models to recapitulate patient’s response to treatment.

## Material and methods

### Tumor sample

#### Ethical considerations and regulatory aspects

Fresh tumoral tissue from an ovarian clear cell carcinoma was collected from a patient treated at the Comprehensive Cancer Center Francois Baclesse (Unicancer Center, Normandy) by the Biological Resources Center ‘OvaRessources’ (NF-S 96900 quality management, AFNOR No. 2016: 72860.5). The biological collection was declared to the French Ministry of Education, Health and Research (No. DC 2010–1243). Informed consent form was signed by the patient and was obtained under the agreement of the ethical committee “North-West III” (CPP). Clinical treatments and histopathological details were extracted from patient charts.

#### Processing of sample

Tumor tissue was cut into 4mm^3^ pieces, which were randomly assigned to the analysis described below. One piece was fixed in 3% paraformaldehyde for paraffin inclusion and histopathological/immunochemistry analyses, two pieces were snapped frozen in FlashFreeze (Milestone) and stored at -150°C for DNA/RNA extractions, three pieces were dedicated to PDX establishment, and another piece was dissociated using the Tumor Dissociation human kit and the gentleMACS Dissociator (Miltenyi Biotec) according to the manufacturer's instructions to establish PDTO and cell line models.

### Establishment of patient-derived models

#### PDX establishment

Immediately following patient’s surgery, tumor fragments were subcutaneously engrafted into the scapular area of anaesthetized nude mice. Tumor growth was measured twice a week and serial fragment grafts of each tumor were conducted on 3 to 5 athymic nude mice. When the tumors reach a volume of 800 to 1000 mm3, tumors were harvested, one fragment was fixed in 3% paraformaldehyde for paraffin embedding and histopathological/immunochemistry analyses, two pieces were snapped frozen and stored at -150°C for DNA/RNA extractions and three pieces were used for passage, residual fragments were frozen in 10% (v/v) dimethylsulfoxid (DMSO) and 90% (v/v) fetal bovine serum (FBS).

#### PDTO establishment

Patient-derived tumor organoids O (derived from the original tumor) and XO (derived from the PDX) were obtained from tumor dissociated cells as previously described [19]. Cells were collected in Organoid Basal Medium (OBM: Advanced DMEM (Fisher Scientific), 10 UI/mL penicillin, 10 µg/mL streptomycin, 1% GlutaMAX-1 (Fisher Scientific)) and pelleted (430g for 5 min). Cells were then resuspended in organoid culture medium (OBM containing B27 (Fischer Scientific, 200 µL/mL), N-Acetyl-L-cysteine (Sigma, 1.25mM), EGF (Miltenyi, 50ng/mL), FGF-10 (Peprotech, 20ng/mL), FGF-basic (Miltenyi, 1ng/mL), A-83–01 (Peprotech, 500nM), Y27632 (Selleckchem, 10µM), SB202190 (Peprotech, 1µM), Nicotinamide (Sigma, 10mM), PGE2 (Sigma, 1µM), Primocin (InvivoGen, 100 µg/mL), Cultrex HA-R-Spondin-1-Fc 293T (AmsBio, 10% V/V) and Cultrex L-WRN (AMS Bio, 50% V/V)). Then, 50µl drops of 1:1 growth factor-reduced Matrigel®/cell suspension containing 10 000 cells per drops were allowed to solidify on prewarmed 24-well suspension culture plates. After polymerization (37°C, 5% CO2, 15 min), each drop was immersed with 500 µL of organoid culture medium. Medium was renewed twice a week and PDTO were passaged every 2–3 weeks: PDTO were collected with cold OBM supplemented with 1% BSA, centrifuged at 200g for 2 min and incubated with TrypLE Express (Gibco) for up to 10 min at 37°C. After dissociation, cells were centrifuged at 430g for 5 min, resuspended in organoid culture medium and counted. Then, 50 µl drops of Matrigel-cell suspension (10 000 cells per drops) were placed in prewarmed 24-well plates. Upon completed gelation, 500µL of organoid culture medium was added to each well. Plates were then transferred to a humidified 37°C/5% CO2 incubator.

#### Establishment of patient-derived cell lines

*From fresh tumor sample*. Patient-derived cell lines L (derived from the original tumor) and XL (derived from the PDX) were obtained from tumor dissociated cells. Cells were collected, plated and grown in cell line medium (RPMI 1640 (Gibco) medium supplemented with 2 mM Glutamax™, 25 mM HEPES (4-(2-hydroxyethyl)- 1-piperazineethanesulfonic acid), 10% heat-inactivated FBS (Fetal Bovine Serum) (Gibco) and 33 mM sodium bicarbonate (Gibco)).

*From PDTO.* PDTO were allowed to adhere to the bottom of the well and to grow as monolayer in the cell line medium described above.

All patient-derived cell lines were maintained in a 5% CO2 humidified atmosphere at 37 °C and passaged once a week. Cell lines were routinely checked for mycoplasma.

All tumor-derived models (cell lines, PDX and PDTO) were authenticated by comparison of their short tandem repeat (STR) profiles with that of tumor of origin (Microsynth) (Supplementary Fig. 1).

#### Commercially available cell line culture

Human ovarian cancer cell lines SKOV3 and JHOC-5 were obtained respectively from ATCC (LGS Standards, Molsheim, France) and the RIKEN institute (Japan) (RCB 1520). Cell lines were grown in RPMI 1640 (SKOV3) or DMEM (JHOC-5) media, supplemented with 2 mM Glutamax, 25 µM HEPES, 10% fetal calf serum and 33 mM sodium bicarbonate (ThermoFisher Scientific, Illkirch, France). All cell lines were maintained in a 5% CO2 humidified atmosphere at 37 ◦C.

### Evaluation of response to treatments

#### Drugs

After reconstitution in saline solution*,* doxorubicin (Teva) and gemcitabine (Sandoz) were stored at 4°C and carboplatin (Accord) was stored at room temperature. Olaparib and belinostat (Medchemexpress) were diluted in DMSO and stored as stock solution at -80°C.

#### Cell line treatment and real-time cell analysis

Real-time growth curves monitoring was performed with the Real-Time Cell Analyzer Multi-Plate instrument, using the xCELLigence System (Agilent, Ozyme, Saint Quentin en Yvelines, France). This system monitors cellular events in real-time by measuring electrical impedance across interdigitated micro-electrodes integrated into the bottom surfaces of 96-well E-plates VIEW (Ozyme). These electrodes measure CI (Cell Index) based on impedance, this measure correlates with the area of cells attached to the bottom of the plate. The CI values are displayed in the plot. Briefly, the cells were plated in 96-well E-plates VIEW and placed onto the Real-Time Cell Analyzer Multi-Plate located inside a tissue culture incubator. Cells were left to grow for 24h before treatment and impedance was continuously measured until the end of the treatment. Standard deviations of well replicates were analyzed with the RTCA 2.1.0 software (Agilent).

#### PDTO treatment

Response of PDTO to treatment was assessed as previously described [20]. When organoids reached the size of 75-150µm in diameter, PDTO were collected with cold OBM supplemented with 1% BSA and centrifugated at 200g for 2min. PDTO were resuspended in organoid treatment medium (organoid culture medium lacking primocin, Y-27632 and N-acetylcysteine) and counted. PDTO were resuspended in 2% Matrigel/organoid treatment medium and 200 PDTO per well were seeded in 100μL volume in a previously coated (1:1 treatment medium/Matrigel®) white clear bottom 96-well plates (Greiner). Thirty minutes later, organoids were exposed to treatments. For X-ray, PDTO were irradiated using CellRad (Faxitron) before seeding. During the treatment, PDTO morphology was monitored using IncuCyte S3 ZOOM (Sartorius) located in a humidified 37°C/5% CO2 incubator. One week later, ATP levels were measured by CellTiter-Glo 3D assay (Promega) and luminescence was quantified using Centro XS3 LB 960 (Berthold Technologies, Bad Wildbad, Germany) with Miko Win 2000 software. Cell viability values were normalized to control and treatment sensitivity was expressed as the average of two independent biological replicates. Viability curves were designed using GraphPad Prism software (version 9.2.0). The half-maximal inhibitory concentration (IC50) was computed for each PDTO model.

#### Reference PDTO

To assess O and XO sensitivity or resistance to the different treatments, results were compared to a collection of other ovarian PDTO for conventional treatments, olaparib and belinostat, or compared to head and neck squamous cell carcinoma PDTO for X-ray sensitivity. One or two references PDTO per treatment were displayed.

#### PDX treatment

PDX fragments were subcutaneously implanted into nude mice as described above. On the first day of treatment, the animals bearing 100 to 200 mm^3^ tumors were randomly distributed to the various treatment and control groups (8–10 mice per group). Carboplatin (Accord), doxorubicin (Teva) and gemcitabine (Sandoz) were diluted in 0.9% sodium chloride. Drugs were administered intraperitoneally at the following regimen: carboplatin 50 mg/kg once a week for 4 weeks; doxorubicin 2 mg/kg once a week for 4 weeks; gemcitabine 80 mg mg/kg once a week for 4 weeks. Mice were weighed and tumor volumes were determined once or twice weekly from two-dimensional caliper measurements using the equation: Tumor volume (mm^3^) = [length (mm) x width (mm)^2^]/2. After 28 days of treatment, mice were euthanized and tumors were harvested for analysis. Tumor growth inhibition (TGI) was calculated using the formula [(median *T*_DayY_ − median *T*_DayX_)/(median *C*_DayY_ − median *C*_DayX_)] × 100 (where DayY is the day of evaluation, and DayX is the day of initiation of therapy for treated [*T*] and control [*C*] tumor volumes). TGI was evaluated according to NCI standards, a TGI ≤ 42% being the minimal level to declare antitumor activity (inactive > 42%, active ≤ 42%, − 10% < TGI ≤ 10%, corresponding to a tumor stabilization, ≤  − 10% and/or partial or complete regressions, corresponding to cytoreductive antitumor activity) [21]. These experiments were performed under guidelines from the European Community Council (2010/63/EU) and approved by the protocol APAFIS #9577 validated by the French ethics committee “Comité d’éthique de Normandie en matière d’expérimentation animale” (CENOMEXA).

### Characterization

#### Histology and immunohistochemistry

Tissue and PDTO were fixed in 3% paraformaldehyde overnight. After embedding PDTO in 2% agarose, tissue and PDTO were dehydrated, paraffin embedded, and sectioned before standard hematoxylin and eosin staining (H&E). Automated immunohistochemistry using a Ventana Discovery XT autostainer (Roche) was performed on 4 µm-thick paraffin sections. Slides were deparaffinized with EZPrep buffer and epitopes were unmasked by 15 min of high-temperature treatment in CC1 EDTA buffer. Sections were incubated for 40 min at 37°C with an anti PAX8 (ab191870, Abcam, 1/500), p53 (ab16665, Abcam, 1/100), WT1 (ab89901, Abcam, 1/300), HNF1β (ab213149, Abcam, 1/2000), Ki67 antibody (NCL-Ki67p, Novocastra, 1/500) or Napsin A (ab133249, Abcam, 1/2000). Secondary antibody (Omnimap Rabbit HRP; Ventana Medical System Inc., Tucson, AZ, USA) was incubated for 16 min at room temperature. Immunodetection performed without the primary antibody was used as control. After washes, the staining was performed with DAB (3, 3'-diaminobenzidine) and sections were counterstained with hematoxylin using Ventana reagents according to the manufacturer's protocol. Stained slides were then digitized using an Aperio ScanScope slide scanner (Aperio Technologies).

#### GIEMSA staining

Cell lines were grown to a confluence of approximately 80% in 6-well plates (Eppendorf). Medium was removed and cells were rinsed with ice-cold PBS (phosphate-buffered saline). Cells were fixed with 70% ethanol for 1h at 4°C and washed three times with deionized water. Cell lines were then stained using a solution of 25% Giemsa (Sigma-Aldrich) diluted in deionized water, washed with 70% ethanol twice and deionized water until purple stain of the solution disappears.

#### Transmission electron microscopy

PDTO were cultured on microscope coverslip. The PDTO were fixed with 2.5% glutaraldehyde in cacodylate buffer 0.1M pH 7.4 overnight at 4°C and rinsed in cacodylate buffer 0.1M pH 7.4. They were then post-fixed 1 h with 1% osmium tetroxyde in cacodylate buffer 0.1M pH 7.4 at 4°C and rinsed in cacodylate buffer 0.1M pH 7.4. The PDTO were then dehydrated in progressive baths of ethanol (70–100%) and embedded in resin Embed 812. After 20h of polymerization at 60°C the coverslips were then separated from the PDTO’s resin bloc and the polymerization was continued for 28 h. Ultrathin sections were done and contrasted with uranyle acetate and lead citrate. The PDTO were observed with transmission electron microscope JEOL 1011 and image were taken with camera Orius 200 (Gatan) and Digital Micrograph software.

#### Scanning electron microscopy

The PDTOs were grown and processed in the same way as in the transmission electron microscopy protocol until the 100% ethanol dehydration step. PDTO were then critical point dryed (Leica, CPD 030) and were sputtered with platinum (JEOL, JFC 1200) before observation with the electron microscope JEOL 7200.

### Transcriptomic analyses

#### RNA extraction

RNA extractions were performed using the NucleoSpin RNA Kit according to the manufacturer protocol (Macherey–Nagel). After extraction RNA samples were stored at -80°C.

#### Transcriptomic pipeline

Total RNA yield and quality were assessed on the Agilent 2100 bioanalyzer (Agilent Technologies, Massy, France). One color whole Human (072363_D_F_20190204 slides) 60-mer oligonucleotides 8 × 60k microarrays (Agilent Technologies) were used to analyze gene expression. cRNA labeling, hybridization and detection were carried out according to supplier’s instructions (Agilent Technologies). For each microarray, Cyanine 3-labeled cRNA were synthesized with the low input QuickAmp labeling kit from 50ng of total RNA. RNA Spike-In were added to all tubes and used as positive controls of labelling and amplification steps. The labelled cRNA were purified and 600ng of each cRNA were then hybridized and washed following manufacturer’s instructions. Microarrays were scanned on an Agilent G2505C scanner and data extracted using Agilent Feature Extraction Software© (FE version 10.7.3.1). Microarray data are available through the GEO depository from NCBI (accession no. GSE237277). Normalization, filtering and statistical comparisons were achieved with the limma R package.

#### Analysis of differential expression

We wished to evaluate the differences in gene expression between groups of samples (O, X, XO vs L, XL, OL, XOL). To that end, for any given gene, it was considered differentially expressed if the fold-change in expression was at least |2| in all samples of a group versus all samples in the other groups. We then identified the most perturbed pathways (Gene Ontology – Biological Processes) associated to the differential expression by using GSEA online tool MSigDB [22].

## Results

### Case history

**Fig. 1:**
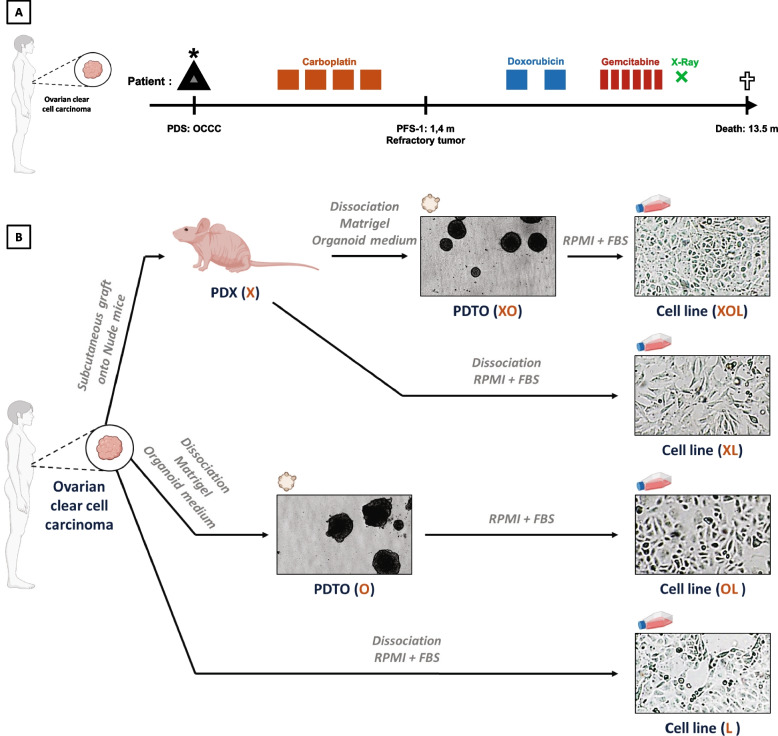
Generation of tumor models derived from the same ovarian clear cell carcinoma (OCCC) patient tumor. **A** Timeline of the medical history of a patient with OCCC. PDS: Primary debulking surgery. PFS: Progression-free survival. **B** Schematic illustration of the process of establishing PDX, PDTO and cell lines from the same OCCC patient tumor

A 57-year-old woman with no significant medical history presented abdominal pain. A computed tomography (CT) scan and a resonance magnetic imaging (MRI) demonstrated ovarian masses with pathological nodes. She underwent staged surgical management with pelvic posterior exenteration, omentectomy, bilateral pelvic and paraaortic lymph node dissection. Pathological examination revealed a clear cell adenocarcinoma with metastatic node and uterine and rectal invasion, stage FIGO IIIA. The patient received first line carboplatin (4 cycles, every 3 weeks) 6 weeks after the surgery. Paclitaxel was planned but she developed a proven allergy after the first cycle. CT scan after 5 cycles found a pelvic relapse, confirmed with MRI. The positron emission tomography (PET) scan found a peritoneal carcinomatosis and a pulmonary metastasis (proven by biopsy). She was treated by two cycles of doxorubicin every 3 weeks. A re-staging CT scan revealed a disease progression. Two cycles of gemcitabine were done with disease progression on the re-staging CT scan. In front of a refractory disease, the patient received exclusive palliative care including X-Ray and died 430 days after the ovarian cancer diagnosis (Fig. 1A).

### Establishment of models derived from this ovarian clear cell carcinoma

Tumor tissue from the left ovarian mass was collected during primary debulking surgery and sent to the laboratory to establish in vitro and in vivo patient-derived tumor models. In total, 7 different models directly or indirectly derived from the tumor of this patient with OCCC were generated (Fig. 1B). The PDX model (named “X”) was established within 4.6 months. In parallel, fresh tumor tissue fragments were dissociated and subsequently seeded into tissue culture-treated plates to generate, under 2.1 months, a tumor cell line growing as monolayer and named “L”. Alternatively, dissociated tumor cells were cultivated in extra-cellular matrix with a specific medium, and successfully generated a PDTO model named “O”, in 1.3 months. After the establishment of these three models derived directly from the patient tumor, one more PDTO model was derived from the PDX and named “XO”. Then, we generated three cell lines from the X, O and XO models, and named “XL”, “OL” and “XOL” respectively.

### OCCC characteristics are preserved in the derived models

**Fig. 2:**
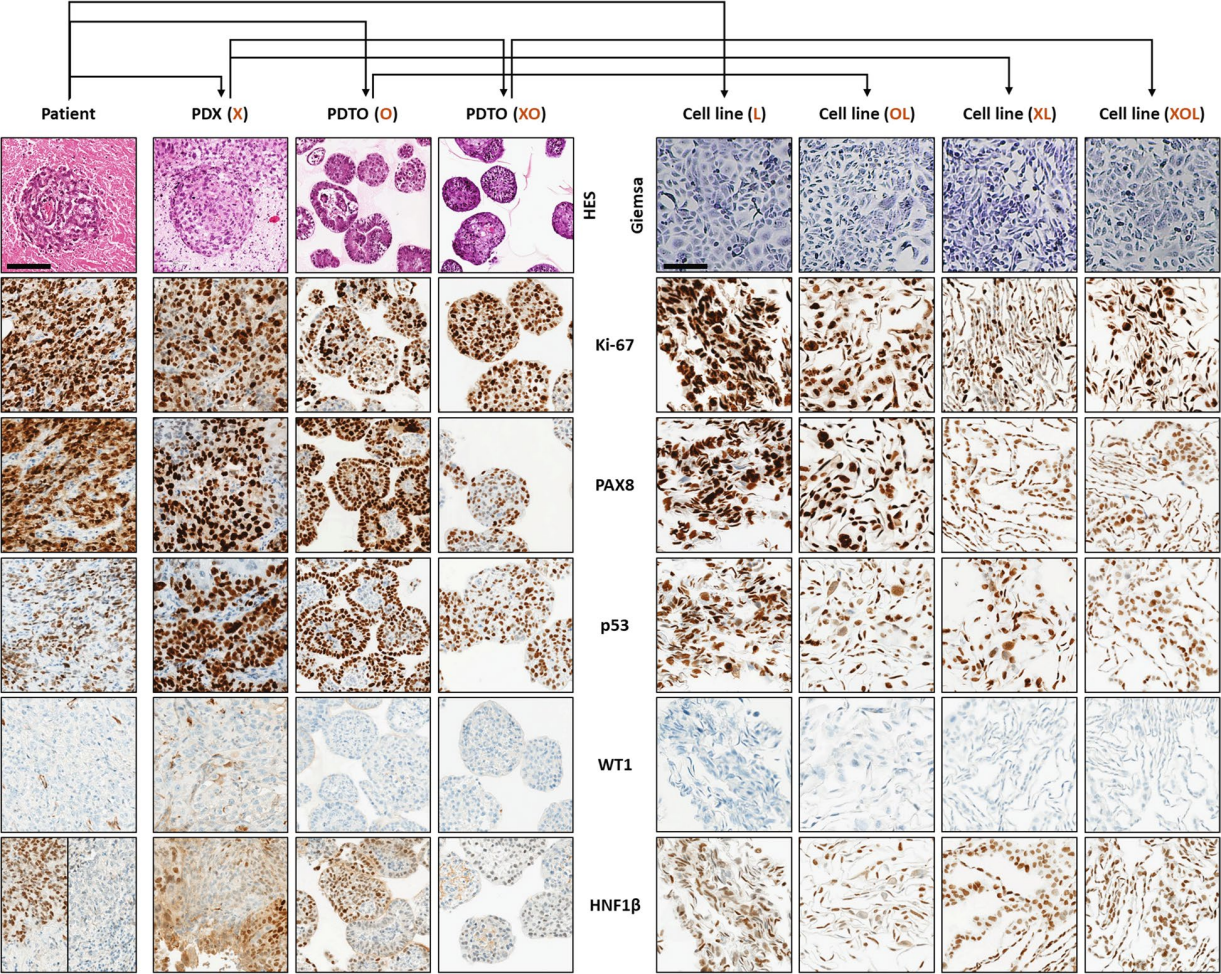
PDX and PDTO recapitulate the heterogeneity of histologic features and expression of OCCC markers of the original tumor. Comparative histologic, morphologic and IHC images of tumor-derived models (PDX, PDTO and cell lines) compared with the original tumor. Top row, H&E staining of patient tumor, PDTOs and PDX and Giemsa staining of tumor-derived cell lines. Next rows, IHC staining of Ki-67, PAX8, p53, WT1 and HNF1B. Scale bar: 100µm

We first studied morphological appearance of the 7 models using H&E or giemsa staining (Fig. 2). The initial tumor displayed large poorly differentiated tumor cells and cells harboring apical hyperchromatic nuclei, also called hobnail cells which are characteristic of OCCC (Supplementary Fig. 2). The tumor also showed large necrosis areas as well as proliferative cells. The PDX model had a structure very close to the one of the initial tumor and also displayed necrotic areas and hobnail cells. The PDTO models O and XO showed a more differentiated architecture with hobnail cells and formation of tubular patterns. Necrosis areas can also be observed in the PDTO directly derived from the patient tumor. Giemsa staining of tumor cell lines showed that the L, OL and XOL cell lines shared an epithelial phenotype. Surprisingly, the cell line XL exhibited elongated shape and a mesenchymal-like phenotype (Fig. 2).

Then, immunohistochemical analysis of a marker panel allowing the diagnosis of clear cell ovarian carcinoma (WT1/p53/PAX8/HNF1-β/Napsin A) and the evaluation of cell proliferation (Ki-67) was performed (Fig. 2). As expected, the initial tumor and all the models harbored nuclear expression of PAX8, a marker for carcinoma of Müllerian origin, and no expression of WT1, a robust prognostic marker selectively expressed in some high-grade serous ovarian carcinoma. Interestingly, p53 was overexpressed in the tumor of origin as well as in the tumor-derived models. However, immunostaining of two OCCC markers Napsin A and HNF1β [23, 24] showed to be discordant in the patient tumor. Napsin A was absent in the patient tumor as well as in the tumor-derived models (Supplementary Fig. 3), by contrast to HNF1β which was heterogeneously expressed in the initial tumor. Taken with the histological analysis, the patient has been diagnosed with OCCC and this diagnosis was confirmed by a histological second opinion of a pathologist expert of the network of rare malignant tumors of the ovary (TMRG) [25]. Interestingly, PDX (X) displayed a similar heterogeneous HNF1β expression pattern, as well as PDTO (O). PDX-derived organoids (XO) expressed HNF1β to a low level (as suggested by the observation of a light nuclear staining), and all the cell lines displayed HNF1β expression.

**Fig. 3:**
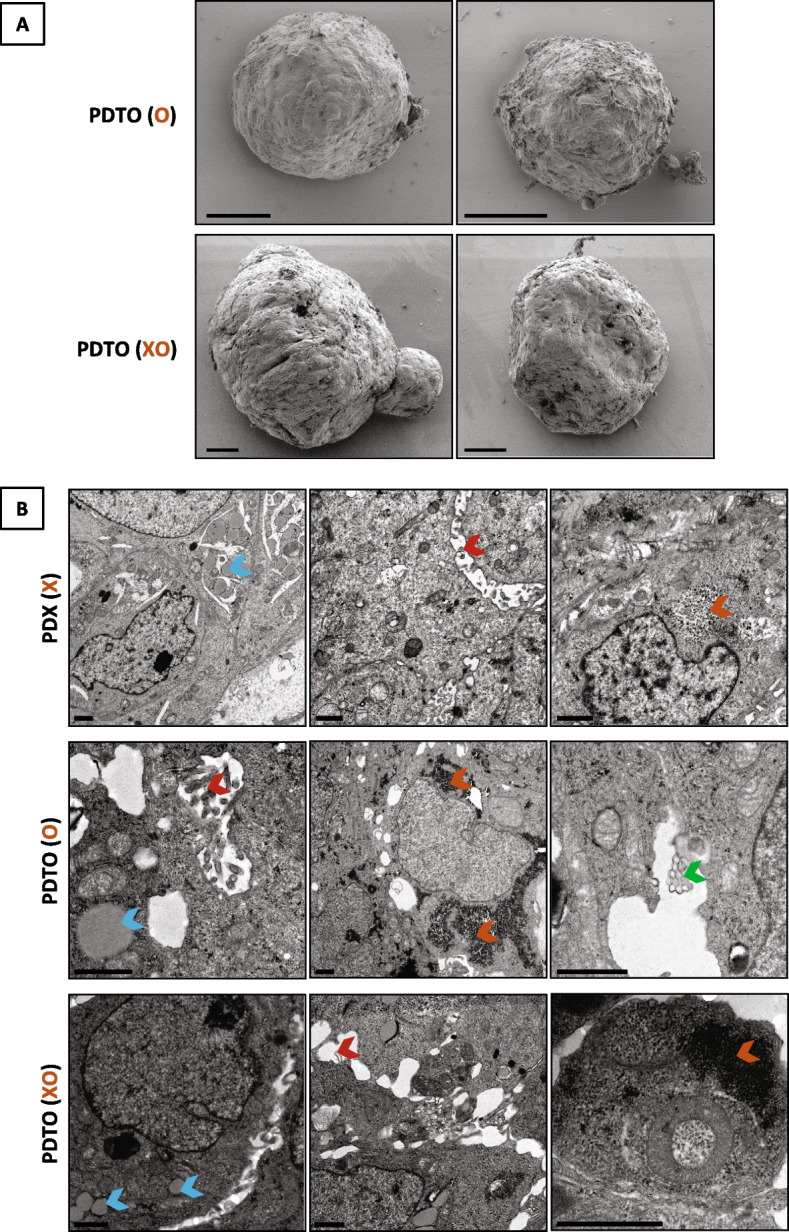
PDTO are highly compacted 3D multicellular structures and display key ultrastructural features of OCCC such as the PDX. **A** Scanning electron micrograph of PDTO derived from the tumor patient (upper panel) or derived from the PDX (lower panel). Scale bar: 20 µm. **B** Transmission electron microscopy images of the PDX (top row), the PDTO derived from the patient tumor (middle row), and the PDTO derived from the PDX (bottom row). Red arrow, microvilli; blue arrow, lipid droplet; orange arrow, glycogen; green arrow, alveolar structure. Scale bar: 1 µm

Morphology studies on PDTO were further investigated using scanning electron microscopy that confirmed that both PDTO were highly compacted (Fig. 3A). Ultrastructure of these well-rounded structures and the PDX was analyzed using transmission electron microscopy. It revealed the presence of key ultrastructural features of OCCC in the three models such as microvilli, lipid droplets, and glycogen with rosette-like appearance. However, these structures are more rarely found in the PDTO XO. Interestingly, alveolar structures were observed only in the PDTO directly derived from the tumor of origin (Fig. 3B).

### PDX and PDTO recapitulate the clinical response of the patient

The potential of the different OCCC models to mimic the clinical situation was evaluated by determining if there was a correlation between the response of the tumor models and the response of the patient to treatments. As previously described, the patient underwent three major therapies: carboplatin, doxorubicin and gemcitabine, and she did not respond to any of them. The 7 models were therefore treated with these three drugs and their sensitivity was assessed.

**Fig. 4:**
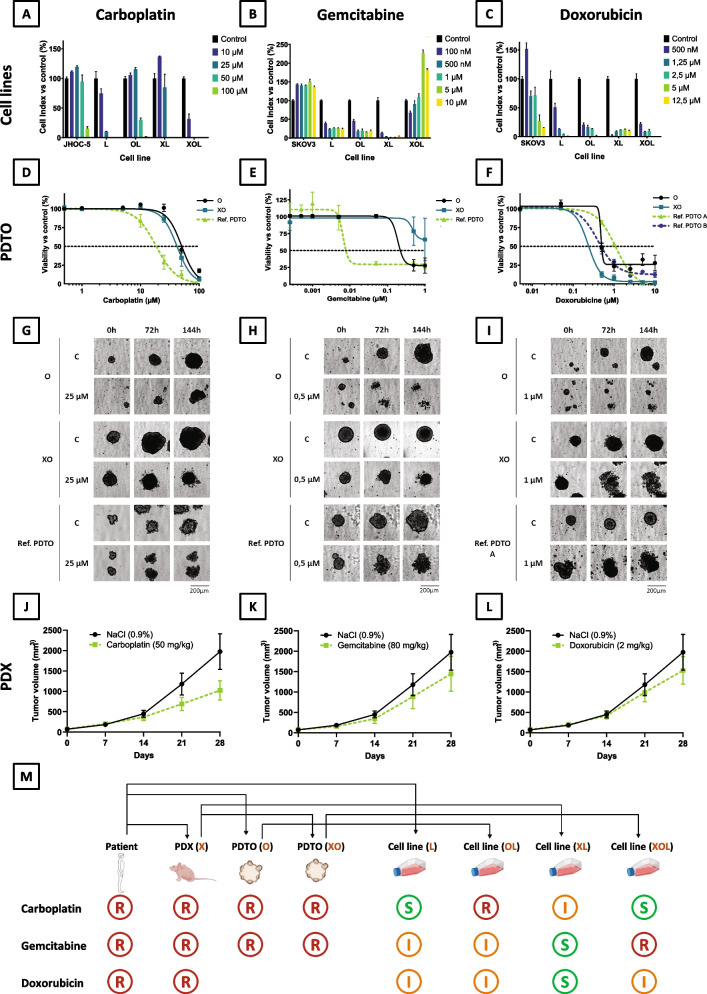
PDX and PDTO recapitulate the clinical response of the patient. **A**, **B** and **C** Cell lines (L, OL, XL, XOL and reference) response to carboplatin, gemcitabine and doxorubicin, respectively. **D**, **E** and **F** PDTO (O, XO and reference) response to carboplatin, gemcitabine and doxorubicin respectively. **G**, **H** and **I** PDTO (O, XO and reference) morphology in response to carboplatin, gemcitabine and doxorubicin respectively at day 0, day 3 and day 6. C = control, scale bar: 200 µm. **J**, **K** and **L** PDX (X) response to carboplatin, gemcitabine and doxorubicin respectively. **M** Summary of the PDX, PDTO and cell lines sensitivity to treatments compared to the patient clinical response

Tumor-derived cell lines L, OL, XL and XOL were exposed to carboplatin (Fig. 4A), gemcitabine (Fig. 4B) and doxorubicin (Fig. 4C), and response was monitored using impedancemetry. JHOC5 OCCC cell line was used as a resistant control for carboplatin and SKOV-3 OCCC cell line for doxorubicin and gemcitabine. According to the calculated cell index after 48h exposure, cell lines exhibit different response profiles, ranging from sensitive to intermediate, with only OL and XOL being resistant to carboplatin and gemcitabine respectively. The XL cell line appeared to be the most sensitive to chemotherapy overall (Supplementary Fig. 4).

PDTO models, were also exposed to carboplatin (Fig. 4D). O and XO displayed IC50 of 50.5µM and 41.9µM respectively, while another sensitive reference PDTO model showed an IC50 of 18.6µM. These data were supported by the PDTO morphological features at the end of the treatment: O and XO grew even at 25µM of carboplatin, while loss of structure of the referent sensitive PDTO was observed at this concentration (Fig. 4G). Similar results were observed after exposure to gemcitabine (Fig. 4E and H), with O and XO being more resistant to these drugs than a sensitive reference PDTO (IC50 of 0.22 µM, > 1 µM and 0.008 µM respectively). For doxorubicin the results are more difficult to analyze since the sensitivity of our tested references PDTO, as well as O and XO models were close to each other’s (IC50 of 1.08 µM for Ref. PDTO A, 0.50 µM for Ref. PDTO B, 0.25 µM for XO and 0.50 µM for O) impeding the discrimination of sensitive from resistant models (Fig. 4F, I and data not shown).

Finally, in vivo tumor growth monitoring of the PDX model treated with carboplatin (Fig. 4J), gemcitabine (Fig. 4K), and doxorubicin (Fig. 4L) revealed modest tumor growth inhibitions (carboplatin: TGI = 56%; gemcitabine: TGI = 52%; doxorubicin: TGI = 75%), TGI ≤ 42% being the minimal level to declare antitumor activity according to the NCI standard [21]. This suggests that the in vivo model was resistant to these treatments (Fig. 4M).

Overall, X, O and XO displayed resistance to the three treatments that were prescribed to the patient, while the four cell lines L, OL, XL and XOL were mostly classified as sensitive or intermediate (Fig. 4M).

Since X-rays have been used only for palliative purpose, the clinical response has not been evaluated (the patient died shortly afterwards). However, O and XO models have been irradiated but displayed a radiation resistance as compared to a head and neck reference PDTO, itself radioresistant (Supplementary Fig. 5).

**Fig. 5:**
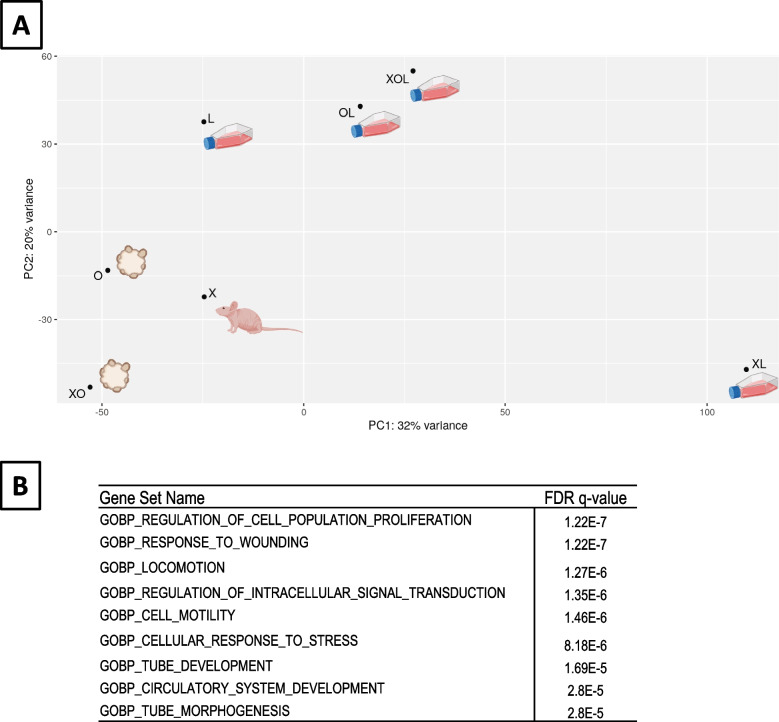
Transcriptomic analysis groups PDX and PDTO away from cell lines. **A** Principal component analysis plot showing groups of samples according transcriptional similarities. **B** Top 10 list of enriched gene sets released by the Molecular Signature Database (MSigDB)

In order to further investigate the differences between the tumor models, a microarray-based transcriptomic analysis was performed on the different tumor-derived models. Due to the advanced stage of necrosis resulting in poor quality of extracted RNA in the original tumor sample dedicated to molecular analyses, this sample could not be included in the transcriptomic study. Principal Component Analysis (PCA) of the microarray data identified a homogenous group of 3 of our models, namely X, O and XO; while all the cell line samples where further away, thus constituting a distinct group (Fig. 5A).

We analyzed the differential expression of genes between these two groups (Supplementary Table 1). Pathways most significantly associated with the differentially expressed genes were related to cell proliferation, cell locomotion, development, and response to stress (Fig. 5B).

### PDTO models are sensitive to Belinostat

**Fig. 6:**
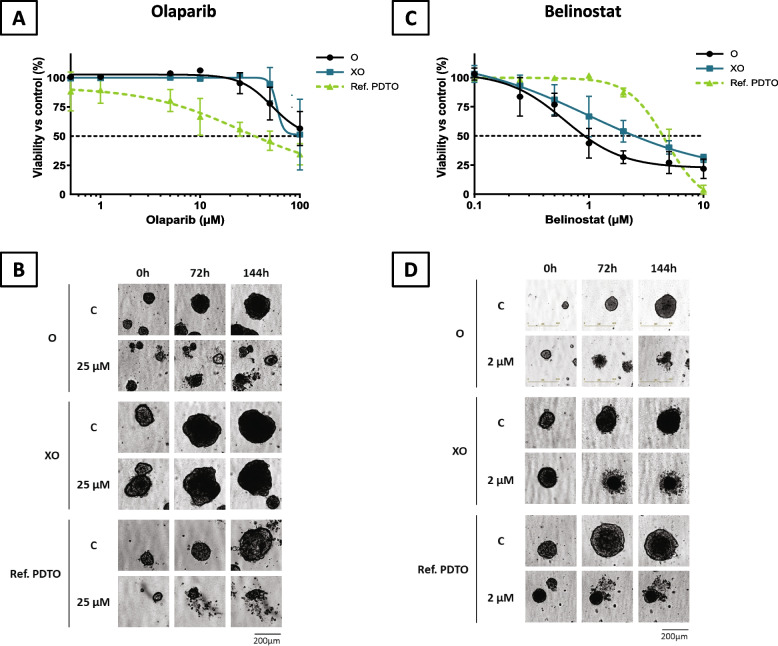
PDTO models are sensitive to belinostat. **A** PDTO (O, XO and reference) response to olaparib and (**B**) their morphology, C = control, scale bar: 200 µm. **C** PDTO (O, XO and reference) response to belinostat and (**D**) their morphology, C = control, scale bar: 200 µm

Since the patient and the PDTO did not respond to the conventional chemotherapies for ovarian cancer, we assessed the antitumoral effect of innovative treatments, olaparib (a PARP inhibitor) and belinostat (a HDAC inhibitor), in the two PDTO models, O and XO. Both O and XO did not respond to olaparib (IC50 unreached at 100µM) while another reference PDTO showed a sensitivity to this inhibitor (IC50 = 35.2µM) (Fig. 6A). PDTO morphology at the end of the treatment supported these data since O and XO structures were intact, even with high concentrations of olaparib (100µM), whereas reference PDTO morphology was highly altered after exposure to 25µM (Fig. 6B). In contrast, O and XO were considered as sensitive to belinostat (IC50 at 0.9µM and 2.5µM respectively) compared to another reference PDTO that displayed an IC50 of 4.4 µM (Fig. 6C and D).

## Discussion

In this study, the objective was to compare the different patient-derived tumor models and tumor of origin in terms of phenotype, histology, molecular features, and response to treatments. All this is obviously of particular interest to define the value of PDTO for the prediction of clinical response and for the development or validation/identification of innovative therapies. This last aspect is particularly relevant since OCCC is a highly aggressive subtype of ovarian cancer associated with resistance to chemotherapy, as observed with the patient from whom these models were derived.

This study reports the establishment and the characterization of 7 models (4 cell lines, 2 PDTO and 1 PDX), all derived from one OCCC tumor including their responses to treatment and their comparison to the clinical response. To our knowledge, this is the first study that compares so many different tumor models derived from the same tumor. Matched PDX and PDTO have been compared to the tumor of origin in panels of colorectal [26] and prostate [27] cancer models as well as PDX and PDTO derived from the PDX (PDXO) in a panel of preclinical models of breast cancer [28] but no comparison with clinical data has been performed. Characterization of models including the PDX, the spheroids generated from the cell lines derived from the PDX and from the tumor (two patients), and the tissue-derived spheres derived from the PDX was also reported in colorectal cancer [29, 30].

The tumor displayed histological features of OCCC with a heterogeneity of HNF1β expression, resulting in a diagnosis of OCCC. This heterogeneity was recapitulated in PDX and PDTO models, but all cell lines displayed a more homogeneous positivity for HNF1β. Analysis of the expression of another marker for OCCC (Napsin A) revealed the absence of this proteins in the patient tumor and the tumor-derived models, questioning the OCCC diagnosis. Nonetheless, several studies have shown that Napsin A was not expressed in all OCCC (only in 80 to 83% of cases [23, 31, 32]). Interestingly, overexpression of p53 was found in tumor of origin and in the tumor-derived models, suggesting p53 mutation [33] despite the fact that such mutation is known to be much less frequent in clear cell carcinoma than in other histological subtypes of EOC [34]. A recent study identified two distinct molecular subclasses of OCCC, including a TP53-mutated group of patients who are more likely to have advanced-stage disease and poorer survival [35], as illustrated by the case history of the patient of this study.

Ultrastructure analysis of PDTO and PDX models revealed the presence of features found in OCCC such as microvilli, lipid droplets and rosette-like glycogen [36]. In accordance with the more undifferentiated phenotype observed in XO model, cells of this model exhibited these differentiation features to a lesser extent. In the PDTO derived from the patient tumor O, alveolar arrangements of tubular structures (honeycomb structures) were observed. Although their exact nature is still unknown, they have been previously described in one case of OCCC [36], suggesting that O model closely recapitulates characteristics of OCCC.

The patient from which all these models were derived was refractory to carboplatin as first line chemotherapy (PFS = 1.4 months) and resistant to the next lines of treatments, doxorubicin and gemcitabine, respectively. In agreement with the clinical response, the PDX model X was resistant to the three treatments delivered to the patient since tumor growth rates are higher than the 42% threshold value at which the drug is considered as effective [21]. The PDTO models O and XO also displayed resistance to carboplatin and gemcitabine compared to other models of the panel of ovarian cancer PDTO of the laboratory. Unfortunately, we were not able to classify the models as sensitive or resistant to doxorubicin since references PDTO as well as O and XO models displayed comparable IC50 values. As a potential explanation, it is known that doxorubicin has limited penetration in solid tumors, especially in tightly packed tumors [37] and that extracellular matrix (ECM) such as Matrigel contributes to reduced doxorubicin sensitivity [38]. Since all PDTO we used are high-density structures (as revealed by scanning electron microscopy pictures of O and XO models in Fig. 3) and cultured in ECM, we could guess that effects of doxorubicin on cells would be probably more dependent on ECM than intrinsic resistance of the cells. However, we cannot rule out that we do not have sensitive PDTO among our models. Nonetheless, a decent correlation between the response of PDTO/PDX and the clinical response was observed overall.

In contrast, the cell lines displayed a higher sensitivity to treatments compared to PDTO and PDX models. This discrepancy suggests that cell line models are not suitable for predictive purposes and for the development of patient-tailored therapies, as already recognized by the scientific community.

The transcriptomic analysis was also consistent with the response of models to treatment. The models recapitulating faithfully the clinical response (X, O and XO) did group together away from the other classical cell culture models. The differences between these two groups of models at the level of biological processes did reflect the morphological differences between these 2 groups, i.e. pathways associated to cell attachment/migration and morphogenesis are likely altered as a consequence of 2D versus 3D growth conditions. Stress conditions might also reflect growth in different environments. However, our transcriptomic analysis is limited by the absence of the tumor of origin and the absence of replicates. Only further functional studies based on genes expression profiles could enable us to draw more precise conclusions on that aspect. However, this pilot study opens up interesting perspectives that can be pursued in future studies, provided that such many tumor-derived models and the tumor sample of origin can be available.

The PDX model X recapitulated both molecular and functional aspects of the original tumor with a response to treatments similar to the clinical response. This aspect has already been described in the literature [3]. Interestingly, PDTO showed the same ability to retain the main characteristics of the initial tumor and the same correlation with clinical response than PDX. In contrast the cell lines are to be excluded from predictive studies as well as from pre-clinical developments. PDX can be used in the development of new molecules, notably because they allow an access to pharmacokinetic, distribution and toxicity parameters. However, they are time-consuming and expensive to set up and process, with a variable rate of establishment depending on the tumor subtypes, thus limiting their use as predictive models. Nevertheless, in some types of aggressive cancers, the establishment of PDX model can be compatible with the time required for clinical management, as it has already been done in advanced cancers [39, 40] to successfully guide the choice of treatment for second and next lines. However, in our study, the 4.5 months establishment time would have been prohibitive for the use of PDX as a tool for predictive functional assay allowing the selection of a personalized treatment after the first line chemotherapy, despite the aggressive nature of the original tumor.

In contrast, the PDTO model displayed an establishment time (1.3 months) that would have been more compatible with its use as a predictive tool in the context of precision medicine. Furthermore, it could allow the evaluation of a greater number of treatments in a reduced timeframe [41]. Nevertheless, it remains crucial to shorten the time required for the evaluation of the response to treatment as much as possible, especially by decreasing the number of PDTO needed for predictive functional assays through miniaturization of functional assays or even through the use of microfluidic devices. Another interest of PDTO compared to other models is the possibility to establish these models from small quantities of material such as biopsies [42].

In the context of our study, the PDTO displayed no sensitivity to the standard-of-care drugs used to treat OCCC, thus matching the response of the patient of origin. Therefore, we investigated molecules that were not used in the patient clinical management to try to identify a potential treatment. First, we selected olaparib, a PARP inhibitor that is effective in an HRD (homologous recombination deficiency) context and can be prescribed as a first-line maintenance in HGSOC and endometrioid carcinoma. Approximately 50% of HGSOC are estimated to be HRD [43], and studies have also found 26% of OCCC that harbored HR-related genes deficiency [44]. Moreover, a subset of preclinical models of OCCC has been shown to display HDR status and to be sensitive to PARPi [45]. However, in our model, olaparib was not effective, which is not quite surprising, since PARPi sensitivity has been associated with sensitivity to platinum salts-based chemotherapy. Next, we investigated PDTO response to belinostat which is a pan-HDAC (histone deacetylase) inhibitor that could be relevant to treat OCCC since HDAC 6 and 7 are specifically upregulated in this pathology [46]. Belinostat is approved in USA for the treatment of relapsed peripheral T-cell lymphoma [47]*.* This work identified belinostat as a potentially effective treatment that could have been administered to the patient. This observation remains to be confirmed in other models of OCCC, perhaps with consideration of histological subtypes and/or epigenetics profiles of these models in order to establish correlation between these parameters and the response to belinostat. Mostly, this opens interesting perspectives concerning the use of PDTO to identify effective alternative therapeutics to be administered to the patient immediately after the failure of the first-line chemotherapy.

In a context of research of efficient treatments with a direct benefit for the patient it would have been possible to test a larger number of molecules and perhaps to identify several alternative treatment possibilities.

## Conclusions

In conclusion, it is possible to establish various models from OCCC which can then be used in a research context but not all of them are completely in adequacy with the clinical situation, neither in molecular and histological terms nor in terms of response to treatment. PDX and organoids appear to be the most relevant models, but PDTO seem to present all the necessary prerequisites for predictive purposes, as it recapitulates the initial tumor histological features and offer a relevant assessment of sensitivity to treatment in a clinical compatible timeframe.

PDTO could thus constitute relevant tools for therapeutic decision support in the context of these particularly aggressive cancers refractory to conventional treatments. This study opens the way to validation studies on a larger cohort of PDTO and to the implementation of clinical trials aiming at confirming their interest in precision medicine in OCCC.

### Supplementary Information

**Additional file 1: Supplementary Table 1.** List of the differentially expressed genes and results of the enriched gene set analysis associated with Figure 5.

**Additional file 2:** **Supplementary Figure 1.** Short-Tandem Repeat Analysis. (A) Electropherogram of the patient tumor and the tumor-derived models. (B) Comparison of the STR profiles of the patient tumor and the different models.

**Additional file 3:** **Supplementary Figure 2.** Histological features of the patient tumor. (A) Clear cells (x10), scale bar: 200 µm. (B) Blue arrows pointing hobnail cells (x10), scale bar: 200 µm. (C) Necrotic area (x5), scale bar: 200 µm.

**Additional file 4:** **Supplementary Figure 3.** The OCCC marker Napsin A is not expressed in the patient tumor and tumor-derived models. IHC images of Naspin A staining of tumor-derived models (PDX, PDTO and cell lines) compared with the original tumor. Scale bar: 200 µm.

**Additional file 5:** **Supplementary Figure 4.** Impedance profile of tumor-derived cell lines and reference cell lines after exposure to conventional therapies. Real-time curves were monitored by impedance measurement (xCELLigence technology). Tumor cells were seeded into 96-well E-Plates VIEW allowing the measurement of Cell Index (CI) based on impedance. Cell lines (L, OL, XL, XOL and reference) were exposed to increasing concentrations of carboplatin, gemcitabine and doxorubicin and CI was recorded every 2 hours for 72 hours. Morphology of PDTO was observed by a fully automated high-throughput cell imaging system.

**Additional file 6:** **Supplementary Figure 5.** Response to radiotherapy. PDTO (O, XO and reference) response to X-Ray was assessed using viability assay.

## Data Availability

The datasets supporting the conclusions of this article are included within the article and its additional files. All materials will be available upon request through a material transfer agreement.

## Abbreviations

AUC: Area Under the Curve
CT: Computed Tomography
DMSO: DiMethylSulfOxide
ECM: ExtraCellular Matrix
EOC: Epithelial Ovarian Cancers
FBS: Fetal Fovine Serum
FIGO: Federation of Gynecology and Obstetrics
GCIG: Gynecologic Cancer Inter Group
H&E: Hematoxylin and Eosin
HDAC: Histone DeACetylase
HGSOC: High-Grade Serous Ovarian Cancer
HNF1-β: Hepatocyte Nuclear Factor-1 Beta
HRD: Homologous Recombination Deficiency
IC50: Half maximal inhibitory concentration
IHC: ImmunoHistoChemistry
MRI: Resonance Magnetic Imaging
MSigDB: Molecular Signature DataBase
OBM: Organoid Basal Medium
OCCC: Ovarian Clear Cell Carcinoma
PARP: Poly (ADP-Ribose) Polymerase
PARPi: PARP inhibitor
PAX8: Paired Box 8
PDTO: Patient-Derived Tumor Organoid
PCA: Principal Component Analysis
PDX: Patient-Derived Xenograft
PET: Positron Emission Tomography
PFS: Progression-Free Survival
STR: Short Tandem Repeat
TG: Tumor Growth
TP53: Tumor Protein 53
WT1: Wilms Tumor Protein
