# Supplementary figures and images for: Comparative analysis of response to treatments and molecular features of tumor-derived organoids versus cell lines and PDX derived from the same ovarian clear cell carcinoma
Source: J Exp Clin Cancer Res. 2023 Oct 7;42:260. doi: 10.1186/s13046-023-02809-8 (PMC10559504; doi:10.1186/s13046-023-02809-8)

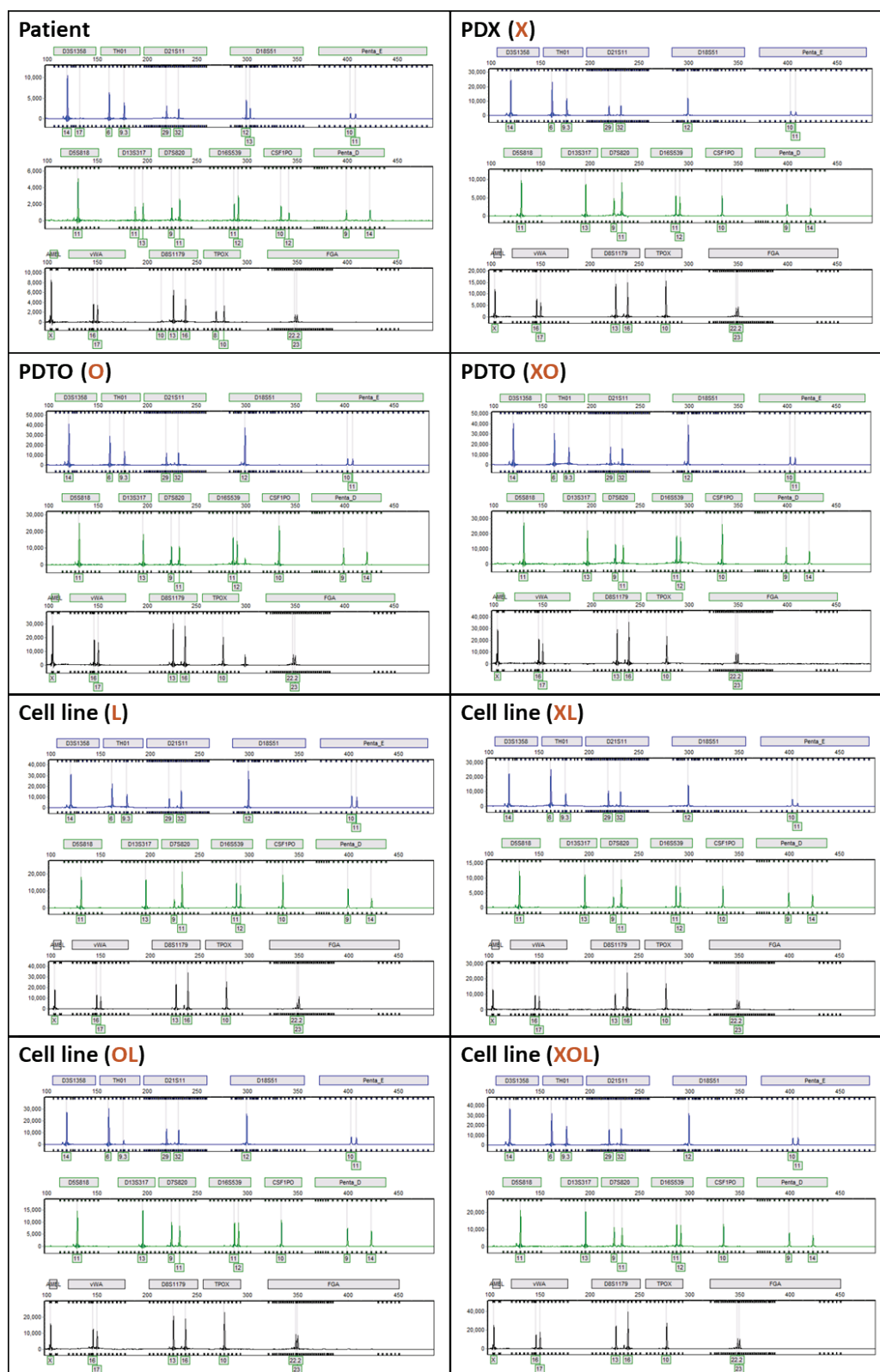[illegible]

Supplement: Supplementary file 2 — Additional file 2: Supplementary Figure 1. Short-Tandem Repeat Analysis. (A) Electropherogram of the patient tumor and the tumor-derived models. (B) Comparison of the STR profiles of the patient tumor and the different models. [file 13046_2023_2809_MOESM2_ESM.pdf]

**A**

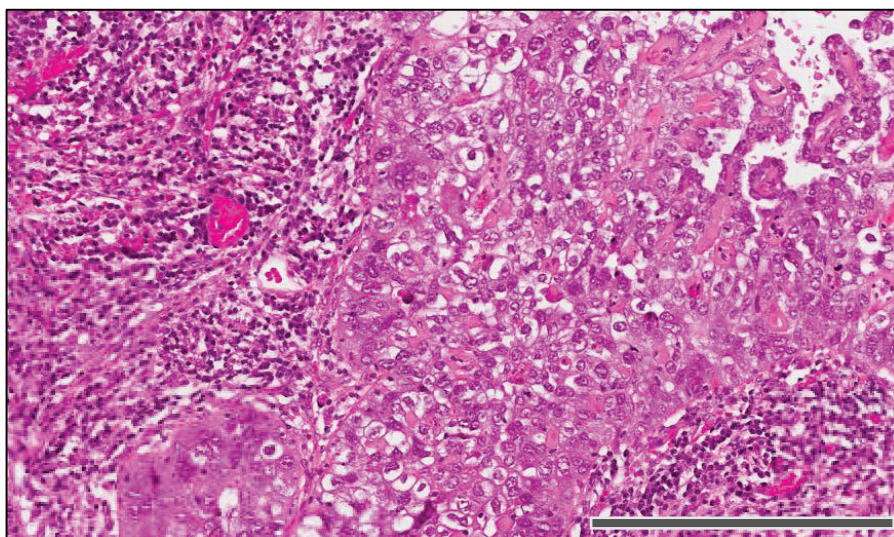

**B**

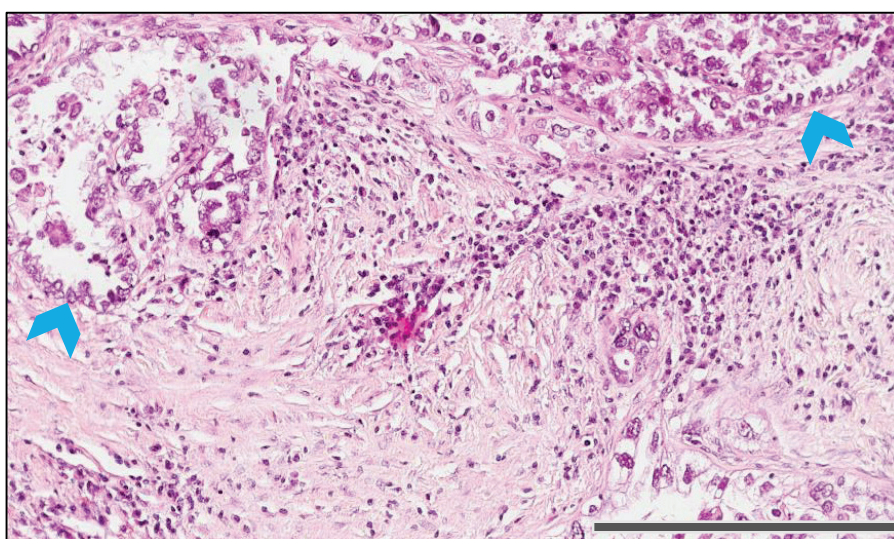

**C**

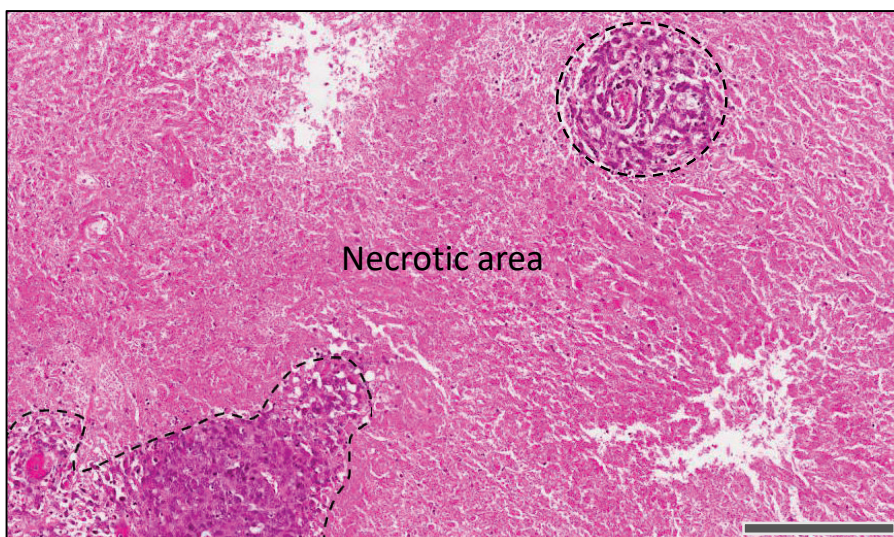

Supplement: Supplementary file 3 — Additional file 3: Supplementary Figure 2. Histological features of the patient tumor. (A) Clear cells (x10), scale bar: 200 µm. (B) Blue arrows pointing hobnail cells (x10), scale bar: 200 µm. (C) Necrotic area (x5), scale bar: 200 µm. [file 13046_2023_2809_MOESM3_ESM.pdf]

Napsin A

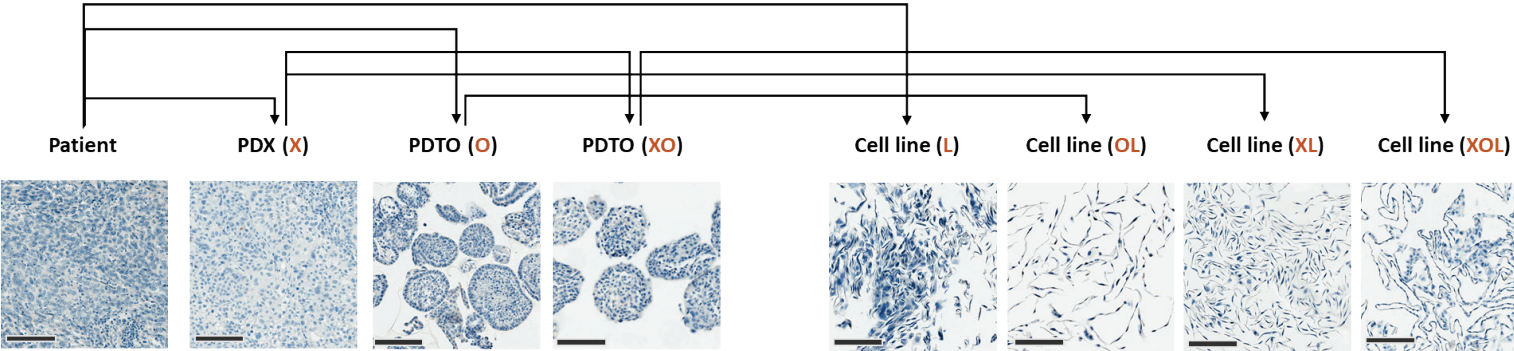

Supplement: Supplementary file 4 — Additional file 4: Supplementary Figure 3. The OCCC marker Napsin A is not expressed in the patient tumor and tumor-derived models. IHC images of Naspin A staining of tumor-derived models (PDX, PDTO and cell lines) compared with the original tumor. Scale bar: 200 µm. [file 13046_2023_2809_MOESM4_ESM.pdf]

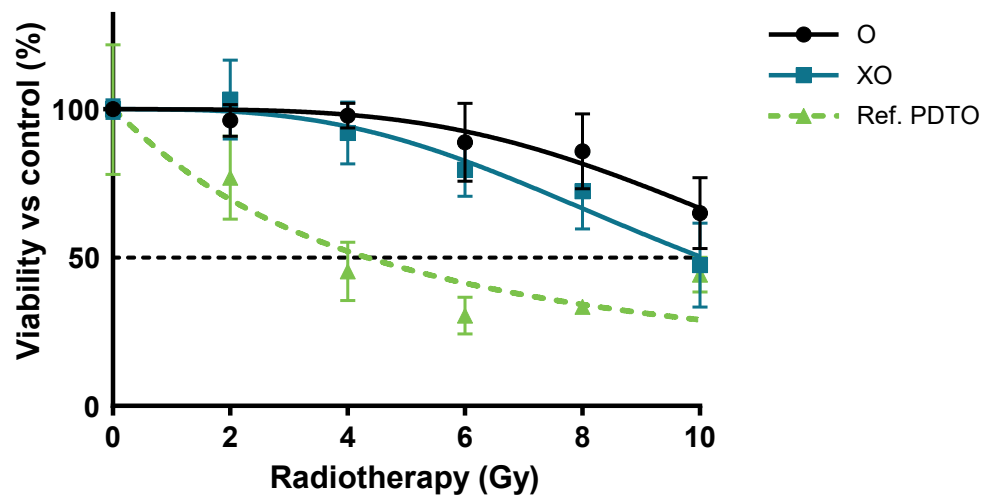

Supplement: Supplementary file 6 — Additional file 6: Supplementary Figure 5. Response to radiotherapy. PDTO (O, XO and reference) response to X-Ray was assessed using viability assay. [file 13046_2023_2809_MOESM6_ESM.pdf]
