# Supplementary material for: Comparative analysis of response to treatments and molecular features of tumor-derived organoids versus cell lines and PDX derived from the same ovarian clear cell carcinoma
Source: J Exp Clin Cancer Res. 2023 Oct 7;42:260. doi: 10.1186/s13046-023-02809-8 (PMC10559504; doi:10.1186/s13046-023-02809-8)
Supplement: Supplementary file 5 — Additional file 5: Supplementary Figure 4. Impedance profile of tumor-derived cell lines and reference cell lines after exposure to conventional therapies. Real-time curves were monitored by impedance measurement (xCELLigence technology). Tumor cells were seeded into 96-well E-Plates VIEW allowing the measurement of Cell Index (CI) based on impedance. Cell lines (L, OL, XL, XOL and reference) were exposed to increasing concentrations of carboplatin, gemcitabine and doxorubicin and CI was recorded every 2 hours for 72 hours. Morphology of PDTO was observed by a fully automated high-throughput cell imaging system. [file 13046_2023_2809_MOESM5_ESM.pdf]

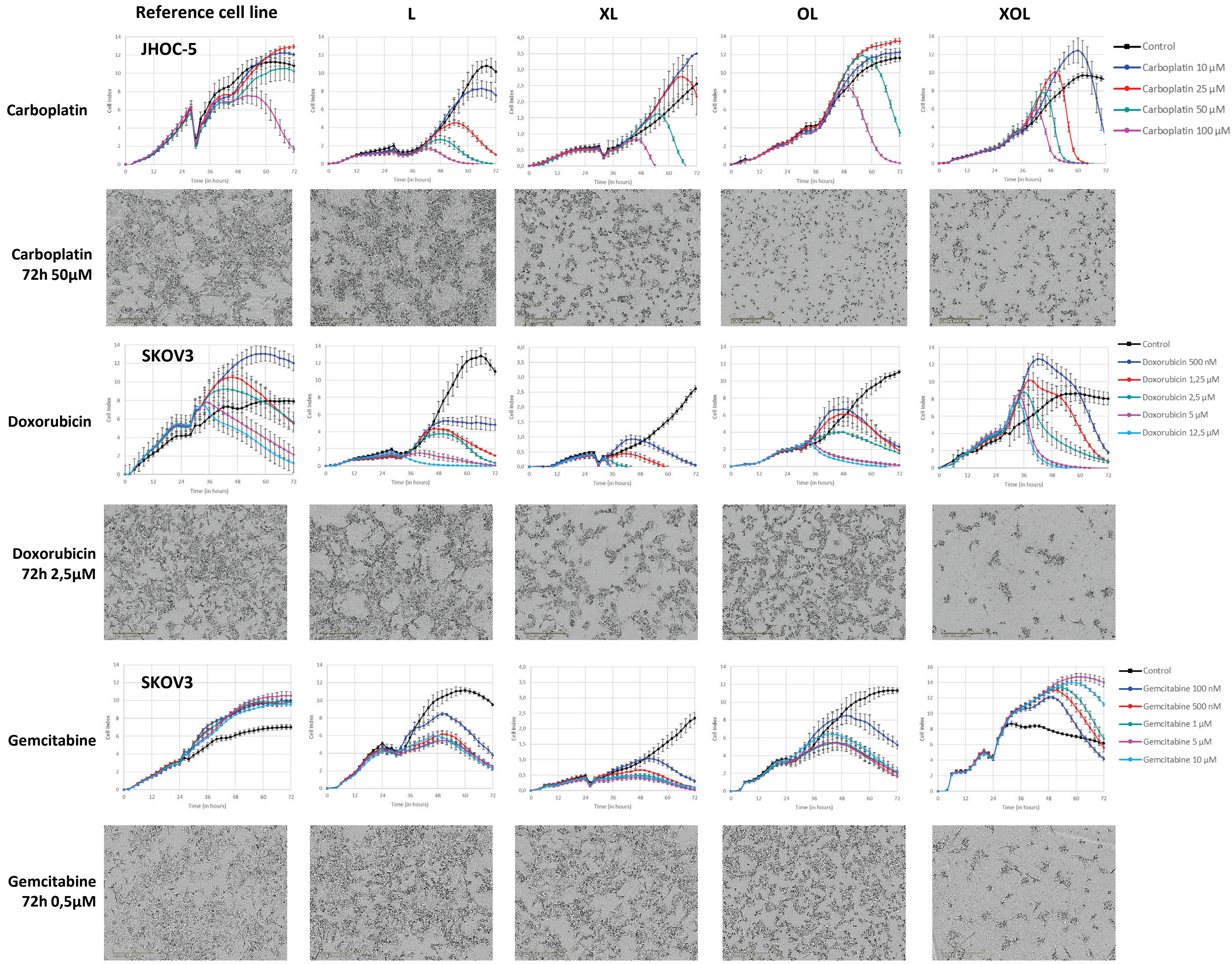

Reference cell line
L
XL
OL
XOL
JHOC-5
Carboplatin
Control
Carboplatin 10 µM
Carboplatin 25 µM
Carboplatin 50 µM
Carboplatin 100 µM
Carboplatin 72h 50µM
SKOV3
Doxorubicin
Control
Doxorubicin 500 nM
Doxorubicin 1,25 µM
Doxorubicin 2,5 µM
Doxorubicin 5 µM
Doxorubicin 12,5 µM
Doxorubicin 72h 2,5µM
SKOV3
Gemcitabine
Control
Gemcitabine 100 nM
Gemcitabine 500 nM
Gemcitabine 1 µM
Gemcitabine 5 µM
Gemcitabine 10 µM
Gemcitabine 72h 0,5µM
Cell Index
Time (in hours)
